# Methods used to address fidelity of receipt in health intervention research: a citation analysis and systematic review

**DOI:** 10.1186/s12913-016-1904-6

**Published:** 2016-11-18

**Authors:** Lorna Rixon, Justine Baron, Nadine McGale, Fabiana Lorencatto, Jill Francis, Anna Davies

**Affiliations:** 1 Centre for Health Services Research, School of Health Sciences, City, University of London, Northampton Square, London, EC1V 0HB UK; 2 Ottawa Hospital Research Institute, Ottawa, Canada

**Keywords:** Fidelity, Receipt, Health intervention, Process evaluation, Implementation

## Abstract

**Background:**

The American Behaviour Change Consortium (BCC) framework acknowledges patients as active participants and supports the need to investigate the fidelity with which they receive interventions, i.e. receipt. According to this framework, addressing receipt consists in using strategies to assess or enhance participants’ understanding and/or performance of intervention skills. This systematic review aims to establish the frequency with which receipt is addressed as defined in the BCC framework in health research, and to describe the methods used in papers informed by the BCC framework and in the wider literature.

**Methods:**

A forward citation search on papers presenting the BCC framework was performed to determine the frequency with which receipt as defined in this framework was addressed. A second electronic database search, including search terms pertaining to fidelity, receipt, health and process evaluations was performed to identify papers reporting on receipt in the wider literature and irrespective of the framework used. These results were combined with forward citation search results to review methods to assess receipt. Eligibility criteria and data extraction forms were developed and applied to papers. Results are described in a narrative synthesis.

**Results:**

19.6% of 33 studies identified from the forward citation search to report on fidelity were found to address receipt. In 60.6% of these, receipt was assessed in relation to understanding and in 42.4% in relation to performance of skill. Strategies to enhance these were present in 12.1% and 21.1% of studies, respectively. Fifty-five studies were included in the review of the wider literature. Several frameworks and operationalisations of receipt were reported, but the latter were not always consistent with the guiding framework. Receipt was most frequently operationalised in relation to intervention content (16.4%), satisfaction (14.5%), engagement (14.5%), and attendance (14.5%). The majority of studies (90.0%) included subjective assessments of receipt. These relied on quantitative (76.0%) rather than qualitative (42.0%) methods and studies collected data on intervention recipients (50.0%), intervention deliverers (28.0%), or both (22.0%). Few studies (26.0%) reported on the reliability or validity of methods used.

**Conclusions:**

Receipt is infrequently addressed in health research and improvements to methods of assessment and reporting are required.

**Electronic supplementary material:**

The online version of this article (doi:10.1186/s12913-016-1904-6) contains supplementary material, which is available to authorized users.

## Background

Health behaviour change interventions are typically complex and often consist of multiple, interacting, components [1]. This complexity is magnified by the fact that these interventions are often context-dependent, delivered across multiple settings, by multidisciplinary healthcare professionals, to a range of intervention recipients [2–4]. As a result, ensuring consistency in the implementation of behaviour change interventions is challenging [5]. Despite this, less attention is given to the implementation of behaviour change interventions than to the design and outcome evaluation of such interventions [6–8].

Intervention fidelity is defined as the ‘ongoing assessment, monitoring, and enhancement of the reliability and internal validity of an intervention or treatment’ [9, 10]. Monitoring intervention fidelity is integral to accurately interpreting intervention outcomes, increasing scientific confidence and furthering understanding of the relationships between intervention components, processes and outcomes [6–10]. For example, if an intervention is found to be ineffective, this may be attributable to inadequate or inconsistent fidelity of delivery by the intervention deliverer, rather than the intervention components or design [10]. This can result in the discard of potentially effective interventions, when in fact inadequate implementation may be responsible (described by some as a ‘Type III error’) [11]. Moreover, assessing fidelity can support the wider implementation of interventions in clinical practice by identifying aspects of intervention delivery that require improvement, and intervention deliverer training needs that may form the basis of quality improvement efforts [3]. The importance of assessing intervention fidelity has been emphasised in the recently developed UK Medical Research Council Guidance for conducting process evaluations of complex interventions [12].

Several conceptual models of fidelity have been proposed, and there is no consensus on how best to divide the study of implementation into key components [13]. Proposed models differ in the number and nature of components argued to represent fidelity. In an attempt to synthesise and unify existing conceptual models of fidelity, a Treatment Fidelity Workgroup part of the National Institute of Health (NIH) Behaviour Change Consortium (BCC) has proposed a comprehensive framework that proposes five components of intervention fidelity: *design*, *training*, *delivery*, *receipt* and *enactment* [9] (see Bellg et al. (2004) [9] and Borrelli et al. (2005) [10] for full definitions of these components). This framework has guided a considerable amount of health research since then [14–17].

The current review examines the methods used to address receipt in health interventions. Patients are now more commonly regarded as active participants in healthcare than as passive recipients [18], particularly with the advent of self-management support in chronic conditions [19]. This active role requires that they engage fully with, understand, and acquire intervention-related skills, so they may subsequently apply them to their day-to-day life (i.e. enactment). As such, receipt is the first recipient-related condition that needs to be fulfilled for outcomes of an intervention to be influenced as intended, and enactment is dependent on this condition being fulfilled.

According to the original BCC framework papers [9, 10, 20], a study that addresses receipt includes one or more strategies to enhance and/or assess participants’ understanding of the intervention and/or the performance of intervention-related skills. The 2011 update [20] added considerations of multicultural factors in the development and delivery of the intervention as a strategy to enhance receipt. Receipt is also defined as the accuracy of participants’ understanding in Lichstein et al.’s (1994) [21] framework, and as ‘ the extent to which participants actively engage with, interact with, are receptive to, and/or use materials or recommended resources’ in frameworks by Linnan and Steckler’s (2002) [22] and by Saunders et al. (2005) [23]. In addition, Saunders et al. (2005) [23] suggest receipt may also refer to participants’ satisfaction with the intervention and the interactions involved. The role of receipt or dose received in these other fidelity, process evaluation, or implementation frameworks, further supports its importance in health research.

Despite this recognised importance of receipt however, systematic reviews to date indicate this concept has received little research attention. Borrelli et al. [10] first examined the extent to which the BCC recommendations to address receipt were followed in health behaviour change research published between 1990–2000. Assessments of participants’ understanding and of performance of skill were found in 40% and 50% of papers, respectively. Strategies to enhance these were found in 52% and 53% of papers, respectively. In subsequent reviews [14–17] the proportion of papers addressing receipt varied between 0% and 79% (see Table 1). In general strategies to enhance receipt have more often been included in studies than assessments of receipt (see Table 1).

**Table 1:** Proportion (%) of papers from past systematic reviews addressing receipt as defined in the BCC framework

Methods for addressing fidelity of intervention receipt	Borelli et al. [10]	Johnson-Kozlow et al. [15]	McArthur et al. [16]	Garbacz et al. [14]	Preyde et al. [17]
1. Assessed participants’ understanding of the intervention	40	52	0	69	30
2. Included a strategy to improve participants’ understanding	52	79	0	66	61
3. Assessed participants’ ability to perform the intervention skills	50	59	50	65	39
4. Included a strategy to improve participants’ performance of intervention skills	53	69	50	66	64
Denominator for proportions presented	325–332^a^	29	10	65	28

Note: ^a^In Borelli et al. [10], the denominator for the proportions provided is the total number of papers for which the method used to address intervention receipt was considered appropriate/applicable by the reviewers, rather than the total number of papers included in the review, i.e. 342. This was 332 for method 1,331 for method 2,326 for method 3, and 325 for method 4

There are limitations to the reviews described above. First, they examined fidelity in relation to specific clinical contexts. Currently there is therefore a need to examine the extent to which receipt has been addressed in the wider health intervention research, a little more than a decade after the publication of the original BCC fidelity framework in 2004 [9]. A second limitation, which also applies to Borelli et al.’s review [10], is that limited attention is given to describing the methods used to address receipt. Comparability and coherence in the methods used across studies is advantageous however, particularly for the effective interpretation and use of systematic reviews in decision-making [13]. Providing a synthesis of fidelity methods used so far would be valuable in guiding future work.

This systematic review was designed to address these limitations. It aimed to describe 1) the frequency with which receipt, as defined in the BCC framework, has been addressed in health intervention studies reporting on fidelity and published since 2004, and 2) the methods used to address receipt. Since receipt is a component in other fidelity frameworks than the BCC, and because it can be reported on in papers without reference to a specific framework, the second aim of this review was broader in scope and examined methods used to address receipt irrespective of whether or which guiding framework was used.

## Methods

### Search strategies

Two electronic searches were used to address the aims of this review. First, to determine the frequency with which receipt, as defined in the BCC framework, has been addressed in health intervention studies since 2004, a forward citation search was conducted using the two seminal BCC framework papers [9, 10]. It was applied to Web of Science and Google Scholar and covered the 2004–2014 period. Results of the second search described below were not used to address this aim, as the focus in search terms on receipt would have introduced bias towards papers reporting on this fidelity component.

Second, to identify methods used to assess receipt in the wider literature (i.e. without focus on the framework(s) used), results from the forward citation search described above were combined with those of a second search performed in five electronic databases (CINAHL, Embase, PsycINFO, Medline, and Allied and Complementary Medicine) using four groups of terms. These comprised synonyms of: i) fidelity, ii) intervention, iii) receipt, and iv) health (Table 2 for a complete list of search terms). Within each group of synonyms, terms were combined using the OR function, and each group of synonyms was combined using the AND function. Terms for receipt and health were used as search terms in all fields (e.g. title, abstract, main body of article), whereas terms for fidelity and intervention were restricted to those contained in titles and abstracts, so as to increase the specificity of the search and identify studies whose main focus was to report on intervention fidelity.

**Table 2:** Search terms

Applied to Titles/Abstracts		Applied to all fields	
Fidelity	Intervention	Receipt	Health
fidelity	Intervention	Recei*	Health
Integrity	Treatment	Enact*	Illness
Intervention quality	Program*	Cognitive skill	Disease
Intervention delivery	Therapy	Behavio* skill	
Intervention implement*		Pa* knowledge	
Treatment differentiation		Pa* acquisition	
Reproducib*		Pa* understand	
Replic*		Pa* comprehen*	
Process evaluation		Repeat	
		Rehear*	
		Behavio* Practi*e	

Note: This search was run in March 2014. Terms within columns were combined using the OR function; groups of synonyms (i.e. columns) were combined using the AND function

*Truncated

### Paper selection

Papers published in English since 2004, and reporting data on receipt of a health intervention were included in this review. A full list of inclusion and exclusion criteria, applicable to results from both searches conducted, is presented in Table 3. These were applied first at the title level, and abstract, and then at the full-text level. They were piloted by the research team on 80 papers and Cohen’s Kappa [24] was k = 0.82. They were refined as appropriate and verified on a further 40 papers. Discrepancies in screening outcomes were discussed until agreement was reached.

**Table 3:** Inclusion and exclusion criteria

Inclusion criteria
• Published since Bellg et al. [9]
• Fidelity of receipt of a health Intervention is assessed (authors had to address receipt using the BCC framework definition of receipt, or had to explicitly refer to other methods used to assess ‘receipt’ or terms considered synonymous such as ‘dose/intervention received’, ‘responsiveness’, or ‘receptivity’).
Exclusion criteria
• Not in English
• Conference or dissertation abstract
• Published before 2004
• Not a health intervention
• Not about fidelity: The paper does not report intervention fidelity; the study may include potential measures of receipt, but it is not clearly related to fidelity
• No data on fidelity: The paper is about intervention fidelity, but it does not aim to present data about fidelity assessment (e.g. protocols, systematic reviews)
• Another type of fidelity: fidelity of receipt not explicitly assessed, or methods for assessing it are not described, and another type of fidelity is assessed (e.g. design, training, delivery, enactment etc.).

Notes: Exclusion criteria were applied sequentially in the order displayed

### Data extraction

A standardised data extraction form was developed and used to extract data in relation to: i) Study aims, ii) Study design, iii) Recipients/participants, iv) Intervention description, v) Information on receipt (guiding fidelity framework, assessment methods, enhancement strategies, etc.), and vii) Data collection details (e.g. timing of measurement (s), sample involved, reliability/validity, etc.). Data were extracted by one researcher and subsequently verified by a second researcher. A third reviewer was involved in instances where there were disagreements, and these were resolved through discussion.

### Analysis and synthesis

All reviewed papers were examined to investigate how receipt was addressed. This investigation first focused on whether receipt as defined in the BCC framework had been addressed (assessments or strategies to enhance participants’ understanding and performance of skill, and consideration of multicultural factors) and then on any other method reported to assess receipt.

A narrative synthesis of the studies reviewed was performed. The proportion of papers citing the BCC framework and addressing receipt as defined in this framework is first presented, then the frequency at which different methods were used to address receipt in the wider literature is provided.

## Results

**Fig. 1:**
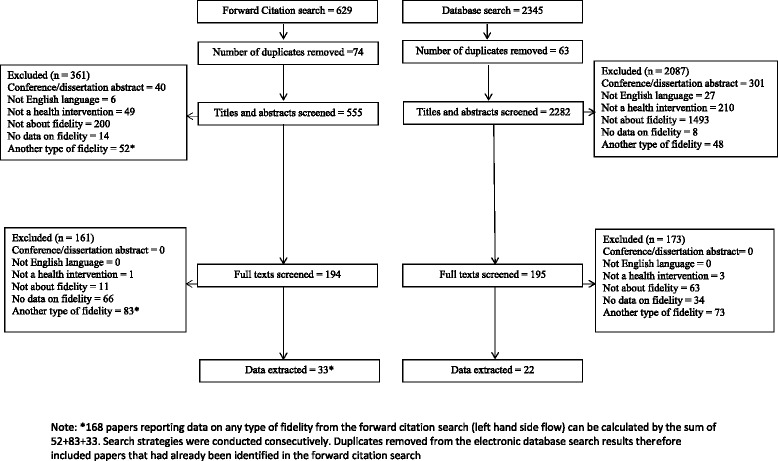
PRISMA Diagram. *168 papers reporting data on any type of fidelity from the forward citation search (left hand side flow) can be calculated by the sum of 52+83+33. Search strategies were conducted consecutively; duplicates removed from the electronic database search results therefore included papers that had already been identified in the forward citation search

A PRISMA flow diagram is presented in Fig. 1. Of the 629 papers identified in the forward citation search, 555 were screened following duplicate removal. Thirty-three of these were found to fit the eligibility criteria for this review and were used to address the first aim of this review.

Of the 2345 papers identified in the electronic database search, 2282 were screened following duplicate removal. Twenty-two of these papers were selected for inclusion in the review. Combined with the forward citation search results, this resulted in a total of 55 papers being used to address the second aim of this review.

A summary of basic study characteristics (study designs, intervention deliverers and recipients, level and mode of delivery) is presented in Table 4 (detailed information on study characteristics available in Additional file 1).

**Table 4:** Summary of characteristics of included studies (*n* = 55)

Study characteristic	*n* (%)		*n* (%)
Design*			
RCT	16 (29.1)	Quasi-experimental	3 (5.5)
Cluster RCT	12 (38.2)	Case study	1 (1.8)
Pilot/feasibility	15 (27.3)	Controlled	1 (1.8)
Pre-post design	2 (3.6)	Unclear	5 (9.1)
Intervention recipients			
People with health conditions	17 (30.9)	Employees/workers	5 (9.1)
Family/informal carers	6 (10.9)	Care home staff and residents	2 (3.6)
Children/Adolescents	9 (16.4)	Ethnic minority women	1 (1.8)
Healthcare staff	9 (16.4)	Smokers (adults and adolescents)	2 (3.6)
Restaurant customers	1 (1.8)	Families	2 (3.6)
People in weight management classes	1 (1.8)		
Intervention deliverers			
Nurses	10 (18.2)	Counsellor/psychologist	2 (3.6)
Allied Health Care professional	7 (12.7)	Academics	2 (3.6)
Organisations (NHS/research council)	2 (3.6)	Teachers	1 (1.8)
Healthcare staff	5 (9.1)	Teachers and Peers	1 (1.8)
Social worker	1 (1.8)	Music therapist	1 (1.8)
Multi-disciplinary team	1 (1.8)	Specialist Trainers	1 (1.8)
Peers	2 (3.6)	Health educators	1 (1.8)
Exercise trainer/physiologist	2 (2.6)	Graduate nurses OR social workers	1 (1.8)
Intervention programme staff	1 (1.8)	Health educator + teacher	1 (1.8)
Team leaders	1 (1.8)	Unclear	12 (21.8)
Level of delivery			
Individual	25 (45.5)	Group	19 (35.1)
Both individual and group	3 (5.5)	Unclear	8 (14.5)
Mode of delivery			
Face to face only	28 (69.1)	Telephone	1 (1.8)
Online (Internet)	2 (3.6)	Telehealth	1 (1.8)
Text messaging	1 (1.8)	Unclear/missing	5 (9.1)
Mixture	7 (12.7)		

Note: *The design is of the study the fidelity assessment is part of, and not the design used to address fidelity

The fidelity research reported was embedded in RCT or cluster RCT designs in most cases (28 studies, 50.9%) but pilot/feasibility designs were also common (15 studies, 27.3%). All interventions included multiple components. The most common components were education or information provision in 19 studies (34.5%) [25–43], and behavioural skills rehearsal or acquisition in 8 studies (14.5%) [25, 26, 30, 38–40, 44, 45]. The largest group of intervention recipients (17 studies, 30.9%) was people with health conditions including adults, women and children [33, 34, 43, 44, 46–58]. It was unclear who intervention deliverers were in 12 studies (21.8%) [26, 39, 46, 50, 51, 55, 59–64], but in studies where this information was identifiable, deliverers were most frequently nurses (10 studies, 18.2%) [33, 35–37, 40, 47, 52, 65–67]. With regards to level and mode of delivery, interventions were most frequently delivered at the individual (25 studies, 45.5%) [27–29, 33, 34, 40, 41, 45, 46, 48, 50–52, 54, 56, 60, 63, 65, 66, 68–73] and group level (19 studies, 35.1%) [26, 31, 32, 38, 39, 42, 43, 49, 53, 55, 58, 61, 62, 64, 67, 74–77]. Face to face was the most common (28 studies, 50.9%) mode of delivery [27, 29, 31, 32, 35–38, 41–45, 49, 50, 56, 58, 60–62, 66–68, 74–78].

### Papers citing the BCC framework and addressing fidelity of receipt as per BCC definition

Of the 629 forward citation search results, 168 papers reported on fidelity of a health intervention (see notes under Fig. 1 to locate these in the PRISMA diagram), 33 (19.6%) of which addressed receipt (studies 1–33 in Table 5). Although all 33 papers cited the BCC framework, 5 (15.2%) papers were not worded in a way to suggest that this framework had informed the fidelity or process evaluation reported [28, 39, 66, 67, 77].

**Table 5:** Methods of assessment and enhancement of fidelity of receipt

Reference (first author)	Intervention recipients	Brief intervention description	BCC definitions of receipt					Other	Methods used to address receipt
			Understanding		Performance of skill				
			Assess	Enhance	Assess	Enhance	Multicultural factors	Other assessment of receipt	
1Asenlof [44]	People with Pain	Individually tailored behavioural medicine intervention				√		Intervention content	Intervention session records collected
									Participants’ individual working sheets collected to examine session content
2Battaglia [65]	People with PTSD	Telehealth tobacco cessation program + PTSD Health Buddy program + motivational interviewing telephone counselling	√		√			Satisfaction	Self-report Questionnaire
3 Blaakman [66]	Caregivers of children with asthma	Tailored nurse led Motivational Interviewing intervention						Engagement	Review of audiotapes and of nurses’ field notes
								Satisfaction	
									Questionnaire to assess satisfaction
4 Black [25]	Adult caregivers	Program to promote caregiver capacity to manage future goals	√				√	Behavioural change	Documentation of changes in care plan
									Verbal verification of understanding of changes
									Self-monitoring of behaviour changes
5 Bruckenthal [47]	Patients with chronic knee pain	Coping skills training for pain	√		√	√		Homework completion	Demonstration and practice of skills reviewed during intervention sessions
6 Carpenter [48]	Adult menopausal women	Deep breathing training and practice supported with CD or DVD	√	√	√	√		Successful attempts to contact participants	Number of participants not reached
									Number of participants requiring media player to play intervention materials
								Availability of hardware to play intervention materials	
									Observation of demonstration of breathing behaviours
								Acceptability	
									Assessment of ability to complete practice log
7 Chee [28]	Caregivers of people with dementia	Skill-building intervention						Contacts with participants	Log of contacts
									Log of problem areas addressed
								Problem areas addressed in intervention	
8 Culloty [75]	Mental health professionals	Cognitive-behavioural therapy supervisor training	√		√			Acceptability	Direct observations (video-taped) of sessions delivered rated by evaluator against Process
									Evaluation of Training and Supervision (PETS) form
									Training Acceptability Rating Scale (TARS) questionnaire
									Focus group interviews (1/7 questions on receipt)
9 Delaney [61]	Homecare professionals	Training on late life depression screening and interventions	√					Self-efficacy (with regards to performance of intervention skills)	Knowledge, self-efficacy, attitude questionnaires in relation to intervention content
								Attitude following workshop	
10 Dyas [50]	Adult patients with difficulty sleeping and healthcare professionals	Training practitioners to deliver problem-focused therapy, patients’ needs and preferences, and sleep consultation video	√					Intervention received	Individual interviews on experiences using intervention, intervention received and understanding
11 Eaton [51]	Adult breast cancer survivors	Web-based cognitive behavioural stress management (CBSM) intervention			√			Use of intervention materials	Website monitoring of chapter completion
									Self reported computer skills
12 Ford [29]	African American, Latina, and Arab women	Education on breast and cervical cancer	√				√		Individual questionnaire items
13 Kilanowski [31]	Children	Education on healthy eating and physical activity	√				√	Attendance	Attendance log
									Knowledge of nutrition/physical activity questionnaire (CATCH)
14 Michie [54]	Adults at increased risk of diabetes	Multi-faceted intervention to increase physical activity			√			Behaviour change and/or maintenance	Self-report (audiotapes)
15 Millear [77]	Adult employees	Strengths-based resilience-building programme.						Receptivity to carrying out intervention skills in daily life	Self-report (questionnaire items)
16 Minnick [67]	Medical practices	The intervention, involved: 1. Joining/forming the team, 2. Assessment, 3. Population focused, care, 4. Process, standardisation,5. Team building, 6. Advanced VIP activities, 7. Ongoing VIP work, 8. Second assessment	√					Accuracy of recall of intervention content (comparison of participants’ recall with deliverers’ recall)	Self-report of intervention content (reports and interviews)
17 Pretzer-Aboff [33]	People with Parkinson’s	Based on social cognitive theory. Aim to increase self-efficacy and outcome expectations, improve physical functioning and activity and ultimately mood and quality of life.	√		√				Direct observation of participants
18 Resnick [36]	Residents and nurses in Assisted Living communities	Intervention components: (1) Environment and policy/procedure assessments; (2) Education; (3). Developing function-focused goals; and (4). Mentoring and motivating	√		√			Perceived effects of exposure to intervention	Focus groups and meetings
19 Resnick [37]	Residents and direct care workers (DCW) in Assisted Living communities	Intervention components: (1) Environment and policy/procedure assessments; (2) Education; (3) Developing function-focused goals; and (4) Mentoring and motivating	√						Self-report of knowledge of intervention content (questionnaire)
20 Resnick [35]	Nursing assistants (NAs) and nursing home residents	Educational programme: sessions addressed the philosophy of restorative care, taught ways to integrate restorative care into daily functional tasks with residents (e.g., bathing, dressing), taught the NAs how to motivate residents to engage in restorative care activities, and defined for the NAs a restorative care interaction and taught them how to document restorative care activities on a daily basis.	√		√				Self-report of knowledge of intervention content (questionnaire)
21 Resnick [34]	Older women post hip fracture	The Exercise Plus Program is a self-efficacy-based intervention to increase exercise. The trainer identifies short- and long-term goals, provides verbal encouragement, and education about exercise	√		√				Direct observation of participants by evaluator using checklist
22 Resnick [56]	Adult stroke patients	Task orientated treadmill based aerobic exercise intervention	√	√	√	√		Attendance	Attendance log
									Direct observation of participants with checklist
23 Robb [57]	Adolescents/young adults (AYA) undergoing stem cell transplant	Therapeutic music video intervention that uses song writing and video production to encourage self-reflection and communication skills	√	√	√	√		Engagement	Active questioning
									Observation of behavioural indicators of participant engagement
24 Robbins [73]	School girls not meeting national guidelines for physical activity	Motivational interviewing counselling sessions to increase physical activity	√					Attendance	Attendance logs
								Engagement	
									Audio recordings of all counselling sessions; content evaluated against checklist
25 Shaw [63]	Adults attending a weight management programme	SMS text messaging intervention to promote sustained weight loss following a structured weight loss programme						Acceptability	Self-report of acceptability on intervention via semi-structured interviews
26 Smith [58]	Patients with type 2 diabetes	Peer support intervention with suggested themes and small structured components						Attendance	Attendance logs
27 Stevens [39]	Rehabilitation team	Rehabilitation team-training intervention to help members of the rehabilitation team gain knowledge and use the new team-functioning skills. Involved: (1) general skills training in team process (e.g., team effectiveness and problem solving strategies) (2) informational feedback (e.g., action plans to address team-process problems and a summary of team-functioning characteristics), and (3) telephone and videoconference consultation (e.g., advice on implementation of action plans and facilitation of team-process skills)	√			√		Active participation in workshop exercises and discussions	Notes and comments based on observation of sessions
									Confirmation of receipt of materials from intervention sites
								Receipt of written intervention materials	
									Self-report in feedback evaluation forms
								Feedback on workshop	
28 Teri [78]	Direct care and leadership staff	Training program designed to teach direct care staff in assisted living facilities to improve care of residents with dementia. Staff are taught to use the activators, behaviours, and consequences (ABC) approach to reduce affective and behavioural problems in residents with dementia by identifying factors within the environment and staff-resident interactions that can altered.	√		√				Checklists and notes
29 Waxmonsky [64]	Providers at community based clinical practices	Standard REP includes an intervention package consisting of an outline, a treatment manual and implementation guide, a standard training program, and as-needed technical assistance. Enhanced REP added customisation of the treatment manual and ongoing, proactive technical assistance from internal and external facilitators.					√	Attendance	Attendance logs
								Contacts	
									Record of intervention contacts (number and length)
30 Weinstein [41]	Women and their live born children	The interventions utilised either brief		√		√		Satisfaction	Self-report in a feedback questionnaire
		Motivational Interviewing							
		(MI) or traditional Health Education (HE) to provide oral health education, assist women to adopt behaviours associated with optimal oral health, and to seek professional dental care for themselves and their young children.							
									Use of standardised protocols/manuals to enhance understanding and performance (no assessment)
31 Yamada [42]	Council members and the health care professionals employed in the NICU	Using knowledge transfer strategies to improve use of pain management strategies in hospitalised infants in neo-natal ICU						Acceptability	Self-report of usefulness of implemented intervention strategies (questionnaire)
									Barriers and facilitators to implementation of intervention strategies (meetings)
32 Yates [43]	Adult CABG patients and spouses participating in Cardiac Rehabilitation (CR)	Patients in both groups participated in the full CR program (comprehensive risk reduction, exercise sessions, and educational classes). Spouses/partners in the PaTH intervention group attended CR with the patient and participated in exercise sessions and educational classes to make the same positive changes in exercise and diet (Therapeutic Lifestyle Change [TLC] Diet recommended by the American Heart Association). Spouses in the usual care group were invited to attend the educational sessions that were part of the CR program.						Attendance	Attendance logs
33 Zauszniewski [45]	Grandmothers who were raising grandchildren	Personal and social resourcefulness skills training.	√		√			Use of skills learnt during intervention	Self-report of use of resourcefulness skills (questionnaire)
									Self-report of skills learnt and used (qualitative- daily journals/voice recordings)
34 Arends [68]	Workers aged between 18–63 years, diagnosed with a common mental disorder.	Evidence-based guideline directed at structuring physicians’ treatment to help sick-listed workers with mental health problems to return to work, using strategies such as problem-solving.						Intervention components completed	Self-report of number of assignments completed (questionnaire)
								Intervention content received	Self-report of topics discussed (questionnaire)
35 Bjelland [59]	11–12 year olds	Intervention aimed at reducing intake of sugarsweetened beverages and sedentary behaviour in adolescent school children.						Exposure	Self-report of awareness of intervention components, receipt of and exposure to intervention materials (questionnaire)
								Satisfaction	
									Self-report of satisfaction (questionnaire)
								Receipt of intervention materials	
36 Boschman [60]	Construction workers	Intervention aimed at detecting signs of workrelated health problems, reduced work capacity and/or reduced work functioning.	√					Recall of intervention-related advice	Self-report (questionnaire)
								Intention to act on intervention advice	
37 Branscum [26]	YMCA-sponsored after school programs	Knowledge and theory-based childhood obesity prevention intervention implemented in afterschool programs. The knowledge-based intervention chose program activities to mediate behaviour change solely based on building awareness and knowledge, such as being aware of the recommended number of servings of fruits and vegetables. The theory- based intervention used theory-oriented program activities to mediate behaviour change such as taking small achievable steps for learning and mastering new skills. Both interventions also included aspects of making and reading comic books “Comics for Health.”						Feasibility	Self-report (questionnaire)
								Acceptability	
38 Brice [27]	Families with recent live births	Infant and child safety focused intervention targeting fire risks, water temperature, electricity, crib hazards, and firearms, as well as potential injuries associated with stairways, pools, and cars. Intervention strategies included the home safety assessment, one-on-one education and counseling, on-site home modifications, further recommendations, and referrals.					√	Satisfaction	Self-report (questionnaire)
39 Broekhuizen [46]	Individuals with familial hypercholesterolemia	Tailored lifestyle intervention aiming to reduce cardiovascular disease (CVD) risk by promoting a healthy lifestyle. Included: improving awareness of CVD risk, motivational interviewing, and computer-tailored lifestyle advice.						Use of (Web) materials	Logins
									Website monitoring of module completion
40 Coffeng [74]	Employees	Group motivational interviewing combined with environmental changes to the physical workplace.						Attendance	Self-report of attendance to intervention sessions and use of intervention components (questionnaire)
								Use of intervention components	
41 Cosgrove [49]	Patients with a primary diagnosis of COPD	Pulmonary rehabilitation programme that provides patients with disease-specific information and teaches self-management skills through the practical application of activities. Includes: educational materials and resources for both health professionals and patients).	√					Acceptability	Self-report of acceptability of intervention (questionnaire)
								Satisfaction	
									Self-report of satisfaction with educational component (questionnaire)
42 Devine [69]	Female employees	Locally adapted obesity prevention intervention involving goal setting, self-monitoring, modelling, and feedback on behaviour.						Intervention content received	Self-report of experiences with intervention and influencing factors (semi-structured interviews and focus groups)
43 Fagan [62]	Youth communities	The Communities That Care (CTC) operating system provides a planned and structured framework for diverse community partners to utilise advances in prevention science. Includes:, (a) assessing community readiness to undertake collaborative prevention efforts; (b) forming diverse and representative prevention coalitions); (c) using community-level epidemiologic data to assess prevention needs; (d) choosing evidence-based prevention policies, practices, and programs and (e) implementing new innovations with fidelity.	√					Responsiveness	Self-report of understanding and participation (questionnaire)
44 Gitlin [70]	Caregivers for patients with dementia	Occupational therapists assess specific needs, concerns, and challenges of caregivers, the physical and social environment, caregiver management approaches, and dementia patient functionality. Involves environmental simplification, communication, task simplification, engaging patient in activities, and stress reduction, and five key treatment principles: client centered; problem solving; tailoring; action-oriented and cultural relevance.	√		√			Adequate number of sessions received and skills learnt	Self-report (questionnaire)
								Treatment received with respect	
45 Goenka [30]	Adolescent Students (6th and 8th Grade)	Intervention involving multiple education sessions, school posters, and parent postcards focused on imparting behavioral skills and contextual knowledge to decrease children’s susceptibility to taking up tobacco in the future.	√				√	Enjoyment and communication skills during intervention delivery	Self-report of enjoyment in teaching, communication skills with participants, ease of use of handbook materials, confidence in using intervention strategies (questionnaire)
								Confidence in using intervention materials and principles	Self-report on participants’ enjoyment, ease of use of materials, participation and absorption (questionnaire)
								Students’ absorption, engagement, participation, ease of use of program materials	
46 Jonkers [52]	Chronically ill elderly patients	Minimal psychological intervention to reduce depression in chronically ill elderly persons involving self-monitoring, exploration of links between cognition, mood and behaviour, and action-planning.						Engagement	Self-report of ability to understand and implement intervention principles (questionnaire)
								Intention to implement intervention	
									Self-report of adherence to previous intervention commitments (checklist)
								Adherence to commitments made	
									Self-report of intention to implement intervention behaviours in daily life (questionnaire)
								Satisfaction	
									Self-report of satisfaction with intervention (questionnaire)
47 Lee-Kwan [71]	Customers of restaurants serving unhealthy foods in deprived areas	A culturally appropriate health eating health promotion intervention in restaurants serving foods high in calories in low-income urban areas.			√		√	Exposure to intervention materials	Self-report of exposure to intervention (survey) examining whether intervention materials were seen and whether this impacted behaviour
								Behavioural change following exposure	
48 Lisha [76]	Adolescent high school Students	A drug prevention programme, with and without combined motivation interviewing.						Attendance	Attendance records
49 McCreary [53]	HIV patients	The six-session intervention was delivered to small groups of 10–12 participants by 85 trained volunteer peer leaders working in pairs						Engagement in group sessions	Observations of group sessions and ratings assigned on indicators of engagement (checklist items)
									Self-report on observations (qualitative comments)
50 Nakkash [55]	Currently married women, aged 18–49, reporting symptoms of medically unexplained vaginal discharge and low to moderate common mental disorders	Psychosocial intervention package targeting the reporting of medically unexplained vaginal discharge and common mental disorders (depression and/or anxiety). Involves progressive muscle relaxation/guided imagery exercises and weekly structured support groups.						Satisfaction	Self-report of participants’ involvement and participation in intervention sessions (questionnaire)
								Level of involvement	Self-report of satisfaction with intervention (questionnaire)
51 Naven [84]	Health visitors	Distribution programmes involved the distribution of free fluoride toothpaste and a toothbrush to all children in Scotland at the age of 8 months, and targeted distribution to ‘at risk’ children aged 1–3 years in areas of deprivation						Receipt of information on intervention requirements	Self-report of receipt of information on intervention requirements (item in survey)
52 Pbert [72]	Adolescent (13-17years) smokers/non-smokers/former smokers	Smoking prevention and cessation intervention tailored to the stage of smoking acquisition of adolescents combined, with peer counselling focusing on the social aspects of smoking and development of the ability to resist social pressures to smoking.						Occurrence of possible intervention steps	Self-report of intervention steps received
53 Potter [32]	Students	Increase children’s exposure to a variety of fruit and vegetables by distributing free fresh or dried fruit and fresh vegetable snacks to all students during the school day. Teachers and school staff were allowed to eat the snacks to serve as role models. Nutrition education and promotion activities were encouraged but not required.						Reactions to program	Self-report of reactions to program (focus groups with separate groups)
54 Skara [38]	Adolescent high school Students	Combined cognitive perception information and behavioural skills curriculum in a high school to prevent drug abuse.						Responsiveness to program	Self-report of responsiveness to program (questionnaire)
55 Teel [40]	Older spouse caregivers of individuals with dementia	Intervention targeting healthy habits, selfesteem, communication, and self-care strategies in older adults. Included practicing healthy habits, building self-esteem, focusing on the positive, avoiding role overload, communicating, and building meaning. Specific self-care strategies were explored in the context of an individual’s experiences, relationships, and condition.	√					Adequacy of communication methods used in intervention	Self-report on helpfulness of intervention to assess understanding of intervention content (interviews)
									Self-report on adequacy of communication methods used (questionnaire)

Twenty-five (75.8%) of these 33 studies addressed receipt in one or more ways consistent with the definitions proposed in the BCC framework. An assessment of participants’ understanding was included in 20 (60.6%) studies [25, 29, 31, 33–37, 39, 45, 47, 48, 50, 57, 61, 65, 67, 73, 75, 78] and an assessment of participants’ performance of intervention-related skills in 14 (42.4%) studies [33–36, 45, 47, 48, 51, 54, 56, 57, 65, 75, 78]. With regards to strategies to enhance receipt, 4 (12.1%) studies reported using a strategy to enhance participants’ understanding [41, 48, 56, 57], 7 (21.1%) to enhance performance of intervention-related skills [39, 41, 44, 47, 48, 56, 57]. Four (12.1%) studies reported having considered multicultural factors in the design or delivery of the intervention [25, 29, 31, 64].

### Methods used to assess receipt

To address the second aim of this review, eligible studies identified through both electronic searches (55 studies) were examined. Information on the methods used to assess receipt in these studies is displayed in Table 5 (further details can be found in Additional file 2).

### Frameworks used

As a consequence of the focus of the forward citation search on the BCC framework, this was the framework used in the majority (28 studies, 50.9%) of studies to inform planning and/or evaluation (i.e. none of the studies included from the electronic database search reported using the BCC framework). Other frameworks that informed the studies reviewed included the process evaluation framework by Linnan and Steckler (2002) [22] in 11 (20.0%) [27, 46, 52, 53, 55, 60, 66, 68, 69, 71, 74], Lichstein et al.’s Treatment Implementation Model (TIM) [21] in 4 (7.3%) studies [28, 39, 40, 67], Saunders et al.’s framework [23] in 5 (9.1%) studies [26, 30, 46, 49, 59], the Reach, Efficacy, Adoption, Implementation, and Maintenance (RE-AIM) framework [79] in 2 (3.6%) studies [46, 70], Dane & Schneider’s framework [80] in 2 (3.6%) studies [38, 76], Dusenbury et al.’s framework [81, 82] in 2 (3.6%) studies [38, 62], Baranowski et al.’s framework [83] in 1 (1.8%) study [52]. A brief definition of how receipt is defined in these frameworks is available in notes below the Table in Additional file 2. More than one of the above frameworks informed the study in 2 (3.6%) of the 55 reviewed studies [46, 52], with a maximum of 3 frameworks being used, none of them being the BCC framework. In 4 studies (7.3%), there was no suggestion that a framework had been considered [32, 72, 77, 84].

### Operationalisations of receipt

Given the focus of the forward citation search on the BCC framework, the two most common ways of assessing receipt in the 55 studies reviewed were measurements of understanding, included in 26 (47.3%) studies [25, 29–31, 33–37, 39, 40, 45, 47–50, 57, 60–62, 65, 67, 70, 73, 75, 78], and of performance of skills, included in 16 (29.1%) studies [33–36, 45, 47, 48, 51, 54, 56, 57, 65, 70, 71, 75, 78].

Receipt was also operationalised in relation to intervention content (e.g. intervention components received or completed, problems areas discussed, advice given) in 9 (16.4%) studies [28, 32, 44, 60, 61, 67–70], satisfaction in 8 (14.5%) studies [27, 41, 49, 52, 55, 59, 65, 66], engagement (level of participation, involvement, enjoyment, or communication) in 8 (14.5%) studies [30, 39, 52, 55, 57, 66, 73, 76], attendance in 8 (14.5%) studies [31, 43, 56, 58, 64, 73, 74, 76], acceptability in 6 (10.9%) studies [26, 42, 48, 49, 63, 75], use of materials (e.g. website use, homework completed) in 4 (7.3%) studies [28, 46, 47, 51], behavioural change and/or maintenance in 4 (7.2%) studies [25, 54, 67, 71], receptivity or responsiveness in 3 (5.5%) studies [38, 62, 77], receipt of intervention materials in 3 (5.5%) studies [39, 59, 84], intention to implement learnings from the intervention in 2 studies [52, 60], telephone contacts during intervention delivery in 2 (3.6%) studies [48, 64], reaction to intervention or feedback on program in 2 (3.6%) studies [32, 39], self-efficacy or confidence in 2 (3.6%) studies [30, 61], exposure (e.g. awareness of intervention) in 2 (3.6%) studies [59, 71], and use of skills learnt in 2 (3.6%) studies [45, 74]. Operationalisations of receipt that were only used in 1 study (1.8%) were attitude in relation to intervention topic [61], perceived effects of exposure [36], treatment received with respect [70], feasibility [26], adherence to commitments made [52], adequacy of communication methods used [40], and availability of hardware to use intervention materials [48].

Studies using the same framework operationalised receipt in many ways, some of which were not consistent with the conceptualisation of receipt proposed in respective frameworks. One example is the 12 studies using the Linnan and Steckler framework [22] in which dose received is defined as ‘the extent to which participants actively engage with, interact with, are receptive to, and/or use materials or recommended resources’. These studies included measures of engagement, present in 4 studies [52, 53, 55, 66] and measures relating to exposure to or use of intervention materials in 3 studies [46, 71, 74], behaviour change following the intervention in 1 study [71], intention to implement intervention in 2 studies [52, 60]. Other measures were used that were less consistent with the frameworks’ definition of receipt. These included measures of satisfaction in 4 studies [27, 52, 55, 66], intervention content in 3 studies [60, 68, 69], attendance in 1 study [74], and adherence to commitments made in 1 study [52].

A second example is the 4 studies using Lichstein et al’s [21] framework in which receipt is defined as the accuracy of participants’ understanding of receipt. These studies included measures of receipt that related to intervention content (problems areas discussed [28], accuracy of recall of intervention content [67]), contacts [28], participants’ receipt of intervention materials [39] or level of participation [39], feedback on the intervention [39], and adequacy of communication methods used [40]. The same applies for studies using other frameworks (see frameworks and measures used in Additional file 2).

### Assessments of receipt

Five (9.1%) studies included only an objective assessment of receipt [43, 44, 46, 58, 76], whilst 7 (12.7%) combined this with a subjective assessment [31, 38, 48, 51, 56, 64, 73]. The majority of studies (43 studies, 78.2%) included only a subjective assessment of receipt (i.e. collected on intervention deliverers or recipients) [25–30, 32–37, 39–42, 45, 47, 49, 50, 52–55, 57, 59–63, 65–72, 74, 75, 77, 78, 84].

### Objective assessments

In the 12 (21.8%) studies that included an objective assessment of receipt [31, 34, 38, 43, 44, 46, 48, 51, 58, 64, 73, 76], this was measured using the number of participants reached during the intervention and the number of participants requiring to borrow hardware to use intervention materials in 1 study [48], website monitoring of module or chapter completion in 2 studies [46, 51], website logins in 1 study [46], records from intervention sessions in 1 study [44], or attendance logs in 8 studies [31, 34, 38, 43, 58, 64, 73, 76].

### Subjective assessments

In total 50 (90.0%) of the 55 studies included a subjective assessment, 21 (42.0%) of which used qualitative methods [25, 28, 32, 33, 36, 39, 40, 42, 45, 47, 50, 52–54, 57, 63, 66, 67, 69, 73, 75] and 38 (76.0%) of which used quantitative methods [26, 27, 29–32, 34, 35, 37–42, 45, 48, 49, 51, 52, 55–57, 59–62, 64–66, 68, 70–72, 74, 75, 77, 78, 84].

Fourteen (28.0%) of the 50 studies included a subjective assessment collected on the intervention deliverer [26, 28, 30, 33, 34, 53, 56, 57, 62, 64, 69, 73, 78, 84], 25 (50.0%) studies on the intervention recipient [27, 29, 31, 32, 35–38, 40–42, 45, 47, 51, 54, 59–61, 63, 65, 70–72, 74, 77], and 11 (22.0%) studies on both of these [25, 39, 48–50, 52, 55, 66–68, 75].

### Assessments collected on intervention deliverers

Twenty-five (45.5%) of the 55 studies that included a measurement of receipt collected this data on the intervention deliverer. Although these were collected on intervention deliverers, they were generally about intervention participants’. An equal number of these assessments involved the collection of qualitative (14 studies, 25.5%) and quantitative data (14 studies, 25.5%). Qualitative data collected in 14 (25.5%) studies consisted of individual interviews, focus groups or reports in 4 studies [50, 52, 67, 69], field notes and comments in 3 studies [39, 53, 66], audio or videotapes of intervention sessions in 3 studies [66, 73, 75], participant observations in 2 studies [33, 48], documentation in participants’ care plan in 1 study [25], records of contacts kept during the intervention in 1 study [28], and active questioning to participants in 1 study [57]. Quantitative data was collected via self-report through questionnaires, surveys or checklists in 8 studies [26, 30, 49, 52, 55, 62, 68, 84], checklists or ratings completed during or following participant observations in 5 studies [34, 53, 56, 57, 78], number and length of phone contacts with participants in 1 study [64].

### Assessments collected on intervention recipients

In total there were 36 (65.5%) studies that included a measure of receipt taken on intervention participants’. Thirteen (23.6%) studies included an assessment of receipt that was performed using qualitative methods. These included interviews in 4 studies [40, 50, 63, 67], focus groups in 3 studies [32, 36, 75], reports in 2 studies [25, 67], audio recordings in 2 studies [45, 54], verbal confirmation of participants’ understanding in 1 study [25], confirmation of receipt of information on intervention requirements in 1 study [39], data on meeting discussions in 1 study [42], and daily journals in 1 study [45], and review of participants’ skills and understanding through demonstrations and practice in 1 study [47]. Quantitative data was collected in just over the majority (29 studies, 52.7%) of studies via questionnaire/surveys [27, 29, 31, 32, 35, 37–42, 45, 48, 49, 51, 52, 55, 59–61, 65, 66, 68, 70–72, 74, 75, 77].

### Validity and reliability of subjective assessments

In only 13 (26.0%) of the 50 studies that included a subjective assessment, there was some consideration made towards the reliability or validity of the methods used to assess receipt [26, 29, 37, 42, 45, 48, 53, 54, 61, 63, 65, 69, 75].

These considerations were reported in relation to quantitative methods (surveys, questionnaires, or checklists) in 10 (26.3%) of the 38 studies making use of these [26, 29, 37, 42, 45, 48, 53, 61, 65, 75]. These considerations included reporting or providing justification for the lack of reporting of Cronbach alpha [45, 48, 53, 65], information on psychometric properties [29, 37, 75], reporting on construct/content validity [42, 61] or on blinding [26].

These considerations were reported in relation to qualitative methods in 4 (19.0%) of the 21 studies using these [45, 54, 63, 69]. Data was coded by more than one person [54, 63], the coder was blinded to group allocation [45], or the scoring attributed to each participant based on the qualitative data collected was calculated independently by 2 researchers and the kappa coefficient for their agreement reported [69].

### Sample selection for receipt assessment

The majority of the 55 studies reviewed (38 studies, 69.1%) [25–30, 33, 35, 36, 38–47, 49, 51, 52, 55–62, 64, 67, 68, 72, 74, 76–78] collected receipt data on all (100%) intervention deliverers’ or intervention participants. There were 4 (7.3%) studies in which the proportion of the sample on which the data was collected varied by assessment measure, one of them being less than 100% [48, 50, 73, 75]. For the 15 (27.3%) studies in which receipt was assessed on less than 100% of the sample, the selection of the subsample assessed was related to missing data or participant withdrawal in 4 studies [63, 65, 66, 70], invitations issued (no further details provided) [50], purposive sampling [54], random selection [56, 73], convenience sampling [53], specific eligibility criteria defined to select the cluster to assess [32], a representative sampling method [69], one in every 5 participants being assessed [71], only one of the intervention groups being assessed [48], or a subset of people randomly selected from one of the clusters assessed [84]. In one study this information was unclear [75].

### Timing of receipt assessments

In 23 (41.8%) of the 55 studies reviewed, the assessment (s) of receipt were conducted during the intervention period (e.g. during/after each intervention session) [25, 27, 28, 30, 33, 34, 43, 44, 46, 47, 50, 54–59, 62, 64, 68, 73, 76, 78]. A slightly lower number of studies (15 studies, 27.3%) included an assessment of receipt that was performed following the intervention [26, 29, 32, 36, 38, 40, 41, 60, 63, 69–72, 74, 77]. Others (14 studies, 25.5%) included assessments of receipt taken at different time points: 4 (7.3%) studies included pre and post assessments [31, 35, 37, 61], one of which combined this with an assessment during the intervention too [31]. Nine (16.4%) studies included assessments taken both during and after the intervention [39, 42, 45, 48, 49, 52, 66, 67, 75]. Another, less frequent combination, consisted in assessments taken before as well as during the intervention, and this was found to happen in 1 study [51]. In 2 (3.6%) studies the timing of the receipt assessments was unclear [65, 84].

Assessments of receipt such as those based on attendance logs, documentation in care plans, field notes, comments, meeting data, recordings, daily journals, observations, records of contacts, demonstrations of skills or completion of practice logs, logins/website monitoring, were generally collected during the intervention period.

Assessments of receipt collected after the intervention were generally those that required participants’ exposure to the intervention, for example measures of satisfaction, acceptability, feasibility, recall of intervention content, feedback forms, use or receptivity to intervention materials/skills, interviews/focus groups on intervention content/experiences using intervention. Assessments based on pre and post intervention measurements were used to examine effects of the intervention on variables such as knowledge or self-efficacy.

## Discussion

The first aim of this review was to identify the frequency with which receipt, as defined in the BCC framework, is addressed in health intervention research. Only 19.6% of the studies identified from the forward citation search to report on fidelity were found to address receipt, compared with 33% in a recent review on clinical supervision [85]. Amongst the studies identified, 60.6% assessed receipt in relation to understanding (compared to 0–69% in other reviews [10, 14–17]) and 42.4% in relation to performance of skill (39–65% in other reviews [10, 14–17]). Strategies to enhance understanding were present in only 12.1% (0–79% in other reviews [10, 14–17]) and performance of skill in 21.1% of studies (50–69% in other reviews [10, 14–17]). These results suggest that there has been little improvement over time with regards to the frequency with which receipt is addressed in health intervention research and that there is a need to continue to advocate for better quality evaluations that focus and report on this fidelity component. These results were further supported in our examination of the wider literature (i.e. not only BCC-related studies), in which understanding was found to be assessed in 47.3% of the 55 studies reviewed and performance of skill in 29.1%. As was suggested by Prowse and colleagues [86], integrating fidelity components to the list of recommended information to report on in reporting guidelines may help increase the proportions of studies addressing and reporting on receipt. Some reporting guidelines have encouraged reporting on fidelity of receipt (e.g. Template for Intervention Description and Replication checklist [87]) but others have not. The Consolidated Standards of Reporting Trials (CONSORT) checklist for RCTs [88] for example emphasises the importance of external validity with regards to generalisability, but the importance of reporting on fidelity is not included. Similarly, a CONSORT extension for non-pharmacological trials [89] does underline importance of reporting on implementation details, but the emphasis is on intervention delivery and not on fidelity of receipt. Consistency across reporting guidelines would help to ensure receipt is addressed and reported more consistently.

The proportions listed above taken from our findings are considerably lower than proportions found in other reviews (see Table 1) that examine receipt using the BCC framework as a guide, particularly with regards to strategies to enhance receipt. Possible explanations for this may be related to differences in the methods used to conduct these systematic reviews. Previous reviews have excluded papers based on study designs. Preyde et al. [17] for example focused only on RCTs and quasi-experimental designs, whilst Garbacz et al [14] required the presence of a comparison or control group. Similarly, McArthur et al [16] included only RCTs and control groups. In contrast, our review was inclusive of all study designs and a considerable proportion was for example, pilot or feasibility studies (27.3%). In a further 5 papers (9.1%) the study design was unclear. Higher quality studies, and those aiming to test hypotheses, may be more likely to monitor and report on fidelity components. Maynard and colleagues [90] for example found that RCTs were 3 times more likely to measure fidelity than studies with a design of lower quality. In this review, studies were not excluded on the basis of study design. We believe that addressing fidelity components is important in study designs like pilot or feasibility studies, and the proportion of these designs included in our review tends to indicate this belief is not uncommon. These trials play a fundamental role in determining the methods and procedures used to assess and implement an approach that will subsequently be used in a larger study and they can help refine an intervention and its implementation to increase its probability of success when evaluated in a larger RCT [91].

Another explanation for some of the differences found between this and other reviews lies in the method used to assess the presence or absence of assessments or strategies to enhance receipt. In other reviews [10, 15–17], fidelity components were judged to be ‘present’, ‘absent (but should be present)’, or ‘not applicable’ (the particular fidelity strategy was not applicable to the paper in question). In this review, the denominator used to calculate proportions was the total number of studies, not only those studies where receipt was deemed to be applicable. It is therefore a conservative estimate of receipt. Similar to Garbacz et al.[14], our review did not account for studies where receipt was not deemed applicable. Performance of a skill, for example, may not have been relevant in all the studies we reviewed. An intervention aiming to provide information on health benefits only (e.g. Kilanowski et al.[31] in this review) is one example of this. As most interventions reviewed involved multiple components and targeted behaviour change, it is unlikely this difference in methods significantly affected our findings. In line with this, future work may benefit from developing guidance for researchers on the types of methods to address fidelity components and that is specific to different intervention types, populations, or evaluation methodologies. Some researchers have begun this process by working towards the identification of features that are unique to the fidelity of technology-based interventions [92].

An important challenge in the field of fidelity is the varying nature of interventions, and the tailoring of the design of an intervention fidelity plan that is therefore required [90]. This is compounded by the other challenge that is the lack of reliable methods available to measure intervention fidelity [93]. The second aim of this review was to describe the methods used to address receipt. Our main findings are that receipt has been operationalised in a variety of ways across studies, and that operationalisations are not always consistent with the framework reported to be guiding the evaluation. Such inconsistencies in the operationalisation of receipt make it difficult to synthesise evidence of receipt and to build a science of fidelity. Clearer reporting of methods to address receipt is also required and may help improve consistency in this field. In this review a third reviewer was involved in data extraction for 18 (32.3%) papers to help reach agreement on the methods used to assess receipt. One common problem was the lack of clear differentiation between fidelity components or other constructs measured and reported on. Ensuring constructs are clearly labelled and differentiated from others is recommended for future work. A recent meta-evaluation of fidelity work in psychosocial intervention research supports our reviews’ findings as it found that there was strong variation in whether authors defined fidelity, that the use of different fidelity frameworks and terminology tended to generate confusion and make comparisons difficult, and that the operationalisation of receipt varied greatly [94]. The BCC framework was an attempt to build consistency in the science of fidelity, but ten years later this attempt does not appear to have been entirely successful. As was underlined by Prowse and colleagues [94] there is a need for standardisation in the field of fidelity, but this must not increase complexity.

A subjective assessment of receipt was included in 90.0% of the studies reviewed, and these were carried out using quantitative (76.0%) and/or qualitative methods (42.0%). Quantitative and qualitative methods have been recognised to provide valuable process evaluation data [13], therefore the combination found in this review is not surprising. One important finding from our review however was that only 26.0% of studies using subjective assessments of receipt reported on the reliability and validity of the measurement tools or qualitative methodology used. More specifically, 26.3% of studies using quantitative methods and 19.0% of those using qualitative methods were found to provide such information. This has been found to be the case in a previous review on fidelity in which none of the studies addressing fidelity were found to have reported on reliability [90]. The lack of information on these issues limits the utility and value of the measures used and their potential to inform evidence-based practice and policy.

### Strengths and limitations of the review

A strength of this review lies in the search strategies used. A forward citation search strategy on the two seminal papers presenting the BCC framework was performed to determine the frequency with which healthcare intervention studies citing this framework assessed receipt. This has been shown to be an effective search strategy to identify literature pertaining to a specific framework or model [95]. Its use in this review was therefore well-suited to the exhaustive identification of relevant papers. Citation searching has been shown to help locate relevant work that traditional database searching sometimes fails to identify [96, 97] but is not commonly used in reviews. The second strategy combined the results from the forward citation search and a database search to examine methods used to assess receipt in healthcare interventions. One other strength of this review is the range of health interventions it covered. Previous reviews on fidelity have focused on specific fields of intervention research and populations (e.g. second-hand smoking [15], mental health [16], and psychosocial oncology [17]. Although Borrelli and colleagues [10] examined a broad range of interventions, their review was published over 10 years ago. To the best of our knowledge, the current review is the first to focus specifically on fidelity of receipt. It was therefore considered more appropriate to broaden the intervention focus as much possible, to reach an overall understanding of the current state of this field of research. Finally, our focus on methods to address receipt has not been investigated before. Earlier reviews [98, 99] have reported on methods to assess fidelity but these were focused on delivery.

This review is not without limitations. First, the first research question focused on the BCC framework. Other fidelity frameworks have been used and the study of their applications may have yielded findings that could have added to our understanding of receipt in interventional research. Despite this we contend that the BCC framework was chosen for its comprehensiveness, as it was developed to unify previously proposed frameworks of fidelity, and to enable comparison with previous reviews that have examined fidelity using this framework. Furthermore, our second research question was broad in scope, and examined the use of several other frameworks. This was to account for the emerging science of fidelity assessment [100], and the likely variability in fidelity conceptualisations and practices.

Second, this review included only published work. The reporting of complex health interventions is often incomplete [101, 102], and the lack of reporting in published manuscripts of fidelity assessments does not necessarily imply their omission from evaluation designs. Consulting the grey literature may have identified a higher frequency with which fidelity of receipt was assessed. Finally, our examination of how receipt was addressed in the literature was applied to the intervention group and not to control groups [20]. We agree that it is important for fidelity to be assessed in control groups, however we did not feel it was within the scope of this review to examine this.

Furthermore, it should also be noted that fidelity of interventions is part of a broader process in which context is an important consideration, in terms of how it affects the implementation of the intervention (e.g. adaptations and alterations to the intervention) and the mechanisms of impact (e.g. participants’ responses to and interactions with the intervention) [13]. For example, in interventions to increase vaccination uptake, both media scares (context) and individual differences in cognitive and emotional antecedents (individual beliefs and fears) to vaccine uptake may be important considerations. If such interventions are not successful in improving participants’ understanding of vaccination, or skills in cognitive reframing regarding vaccination in the context of collective fear, then it is unlikely that vaccination would be enacted and fear would remain. Yet participants with improved understanding and skills in challenging unhelpful beliefs would be more likely to vaccinate. Therefore, for optimal receipt of an intervention, tailoring an intervention to the individual and their social and cultural context will plausibly relate to better receipt of the intervention, which will result in turn improved outcomes. Future studies should examine the extent to which intervention receipt is the mediating mechanism between tailored interventions and enactment, and how these factors impact on outcomes.

## Conclusion

Addressing intervention fidelity is a fundamental part of conducting valid evaluations in health intervention research, and receipt is one of the fidelity components to address. This systematic review examined the extent to which, and the methods used to address receipt in health intervention research in the last ten years. The results indicate a need for receipt to be more frequently integrated to research agendas. The review also identified some issues and concerns relating to the ways in which receipt has been addressed to date, with operationalisations of receipt lacking in consistency. We recommend that information on reliability and validity of the receipt measures be reported in future fidelity research.

Box 1: Lessons learnt and recommendations from this review

**Table:** 

**Lessons learnt**
• Fidelity of receipt (as defined in the BCC framework, i.e. assessments of participants’ understanding and performance of skill and strategies to enhance these) remains poorly assessed in health intervention research
• Reporting of strategies to enhance receipt, i.e. participants’ understanding and performance of skill, remains particularly low.
• Other frameworks than the BCC have been used to guide fidelity/process evaluation work, but operationalisations of receipt do not always match the definitions of receipt provided in these frameworks
• The reporting of methods used to assess receipt requires improvement. Reporting was unclear in a number of papers, requiring readers to read manuscripts attentively several times to identify how receipt was operationalised and providing no information on the validity/reliability of the methods used
• Quantitative and qualitative methods, or a combination of both, have been used to address fidelity of receipt in health intervention research.
**Recommendations for future work**
• In the early stages of study design, consider how to address fidelity of receipt both in relation to assessments and strategies to enhance
• Select one or more fidelity frameworks to guide fidelity work (or use an overarching model) and ensure the methods used to assess receipt are consistent with the definitions of receipt in the chosen framework (s) (provide definitions of receipt)
• Clearly differentiate between fidelity components and other constructs when writing papers (e.g. receipt and enactment are different constructs, therefore methods used to assess them need to be described separately, as well as results).
• Address and report on the reliability and validity of the methods used to assess receipt

## Additional files

Additional file 1: Details of study characteristics. (DOCX 79.9 kb)
Additional file 2: Details on methods used to assess and enhance receipt. (DOCX 91.1 kb)

## Abbreviations

BCC: Change consortium framework
CONSORT: Consolidated Standards of reporting trials
NIH: National institute of health
PRISMA: Preferred reporting items for systematic reviews and meta-analyses
RCT: Randomised controlled trial
RE-AIM: Reach, efficacy, adoption, implementation, and maintenance
TARS: Training acceptability rating scale
Tim: Treatment implementation model
