# Supplementary material for: Methods used to address fidelity of receipt in health intervention research: a citation analysis and systematic review
Source: BMC Health Serv Res. 2016 Nov 18;16:663. doi: 10.1186/s12913-016-1904-6 (PMC5116196; doi:10.1186/s12913-016-1904-6)
Supplement: Additional file 1: — Details of study characteristics. (DOCX 79.9 kb) [file 12913_2016_1904_MOESM1_ESM.docx]

**Additional File 1**. Details of study characteristics

| **Author** | **Design** | **Sample (n)** | **Intervention Recipients** | **Intervention Deliverers** | **Intervention Description** | **Level and mode of delivery** |
| --- | --- | --- | --- | --- | --- | --- |
| 1Asenlof [44] | RCT | 122 | Adults 18-65 years, literate in Swedish, and experiencing persistent musculoskeletal pain | Physical therapists | Individually tailored behavioural medicine intervention; 7 general phases: (1) behavioural goal identification and assessment, (2) self-monitoring, (3) Individual functional behavioural analysis, (4) basic skills acquisition, (5)applied skills acquisition, (6) generalisation, (7) maintenance and relapse prevention | Not reported, face to face |
| 2Battaglia [65] | Pilot (single-group, unblinded) | 11 | Adult veterans with PTSD who smoked | Research nurse | The telehealth care management tobacco cessation curriculum was integrated with the PTSD Health Buddy program and motivational interviewing telephone counselling | Individual, telehealth |
| 3 Blaakman [66] | RCT | 140 | Adult caregivers of smoke-exposed children with persistent asthma | Nurses | Tailored nurse led Motivational Interviewing intervention targeting smoking cessation in caregivers. | Individual, face to face |
| 4 Black [25] | Quasi-experimental  (pre-post tests, no controls) | 120 | Adult caregivers | Social worker | The CARES program (Caregivers Accessing Resources and Essential Services) is to promote caregiver capacity to manage future goals through an increased knowledge of community resources and skills learned throughout the process. | Unclear |
| 5 Bruckenthal [47] | Pilot (single group) | 6 | Patients with chronic knee pain | Nurses | Coping skills training intervention for osteoarthritis pain | Individual, face to face |
| 6 Carpenter [48] | RCT  (3 groups) | 218 | Adult menopausal women | Health care staff | Deep breathing training (laboratory-based sessions) and at-home practice by CD or DVD | Individual, Face to face, & home practice |
| 7 Chee [28] | RCT | 105 | Caregivers for individuals with dementia | Occupational therapists | Skill-building intervention delivered by occupational therapists targeting caregivers. Involved education, problem solving, communication, environmental and task simpliﬁcation techniques, and home modiﬁcations. | Individual home visits and telephone sessions |
| 8 Culloty [75] | Quasi-experimental | 17 | Mental health professionals | Clinical lecturer | CBT supervisor training | Group, face to face |
| 9 Delaney [61] | Pilot (pre-post | 280 | Homecare professionals | Unclear, trainers | A training packet on late life depression screening and interventions | Group, face to face |
| 10 Dyas [50] | Pilot (cluster RCT) | 20 | Adult patients with difficulty sleeping | unclear | Intervention training delivered to practitioners including problem-focused therapy, what patients need and want, and sleep consultation video | Individual, face to face |
| 11 Eaton [51] | RCT | 162 | Adult breast cancer survivors | Intervention delivered via the internet | Web-based cognitive behavioural stress management (CBSM) intervention | Individual (with online group component via the website) |
| 12 Ford [29] | RCT | 514 | African American, Latina, and Arab women | Clinical health workers | Educational sessions on breast and cervical cancer administered in the language of the participants choice; | Individual, face to face |
| 13 Kilanowski [31] | Pilot (single group, pre-post) | 64 | Children | Classroom teacher | Health curriculum programme to teach middle school migrant farmworker children the benefits of healthy eating and activity using a multimedia and ethnic-tailored approach embedded in a 7-week summer Migrant Education Program. | Group, face to face |
| 14 Michie [54] | RCT | 365 | Adults at increased risk of diabetes | Facilitators included a dietician, two nurses and a physical fitness instructor | Intervention programmes are delivered in (i) face to face, and (ii) distance arm. Participants learn strategies to increase physical activity, for instance selecting activities that they enjoy doing, setting achievable goals, defining action plans, self-monitoring, self-reinforcement and relapse prevention. | Individual, face-to-face, telephone and post |
| 15 Millear [77] | Pilot (2 groups, non-randomised, pre-post tests) | 28 | Adult employees | Registered psychologist | The Promoting Adult Resilience (PAR) programme is a strengths-based resilience-building programme that integrates interpersonal and cognitive behavioural therapy (CBT) perspectives. | Group, face to face |
| 16 Minnick [67] | Pilot | 3 | Nongovernmental, urban medical practices | Nurse Coaches | The intervention, involved: 1. Joining/forming the team, 2. Assessment, 3. Population focused, care, 4. Process, standardisation,5. Team building, 6. Advanced VIP activities, 7. Ongoing VIP work, 8. Second assessment | Group, face to face |
| 17 Pretzer-Aboff [33] | Pilot (single group, pre-post tests) | 24 | People with Parkinson’s and their carers | An advanced Parkinson’s nurse | Based on social cognitive theory. Aim to increase self-efficacy and outcome expectations, improve physical functioning and activity and ultimately mood and quality of life. | Individual, face to face. |
| 18 Resnick [36] | Pilot (cluster RCT) | 18 | Nurses in Assisted Living communities | Function focused care nurse | Intervention components: (1) Environment and policy/procedure assessments; (2) Education; (3) Developing function-focused goals; and (4) Mentoring and motivating | Unclear, face to face |
| 19 Resnick [37] | Cluster RCT | 117 residents, 96 care workers | Residents and direct care workers in Assisted Living communities | Two nurse champions | Intervention components: (1) Environment and policy/procedure assessments; (2) Education; (3) Developing function-focused goals; and (4) Mentoring and motivating | Unclear, face to face |
| 20 Resnick [35] | Cluster RCT | 1009 [486 residents, 523 nursing assistants (NAs)] | NAs and nursing home residents | Restorative Care Nurse | Educational programme: sessions addressed the philosophy of restorative care, taught ways to integrate restorative care into daily functional tasks with residents (e.g., bathing, dressing), taught the NAs how to motivate residents to engage in restorative care activities, and defined for the NAs a restorative care interaction and taught them how to document restorative care activities on a daily basis. | Face to face |
| 21 Resnick [34] | Unclear | 205 | Older women post hip fracture | Exercise trainers | The Exercise Plus Program is a self-efficacy-based intervention to increase exercise. The trainer identifies short- and long-term goals, provides verbal encouragement, and education about exercise | Individual, face to face, and telephone |
| 22 Resnick [56] | RCT | Unclear | Adult stroke patients | Exercise physiologists | Task orientated treadmill based aerobic exercise intervention | Individual, face to face |
| 23 Robb [57] | Cluster RCT | Unclear | Adolescents/ young adults (AYA) undergoing stem cell transplant | Board certified music therapists | Therapeutic music video intervention that uses song writing and video production to encourage self-reflection and communication skills | Unclear, Face to face |
| 24 Robbins [73] | Quasi-experimental ( 2 groups, pre-post tests) | 37 | School girls not meeting national guidelines for physical activity | School nurse | Motivational interviewing counselling sessions to increase physical activity | Individual, face-to-face |
| 25 Shaw [63] | Pilot (single group, pre-post tests) | 16 | Adults attending a weight management programme | Unclear, SMS messaging | SMS text messaging intervention to promote sustained weight loss following a structured weight loss programme | Individual, SMS messaging |
| 26 Smith [58] | Cluster RCT | 395 | Patients with type 2 diabetes | Peer supporters | Peer support intervention with suggested themes and small structured components | Group, face to face |
| 27 Stevens [39] | Cluster RCT | 29 | Rehabilitation team | Unclear | Rehabilitation team-training intervention to help members of the rehabilitation team gain knowledge and use the new team-functioning skills. Involved: (1) general skills training in team process (e.g., team effectiveness and problem-solving strategies) (2) informational feedback (e.g., action plans to address team-process problems and a summary of team-functioning characteristics), and (3) telephone and videoconference consultation (e.g., advice on implementation of action plans and facilitation of team-process skills) | Group, face to face, videoconference consultation |
| 28 Teri [78,78] | Pilot (multi-site) | 80 | Direct care and leadership staff | Trainers specialising in dementia care | Training program designed to teach direct care staff in assisted living facilities to improve care of residents with dementia. Staff are taught to use the activators, behaviours, and consequences (ABC) approach to reduce affective and behavioural problems in residents with dementia by identifying factors within the environment and staff-resident interactions that can altered. | Group workshops and individual face to face sessions |
| 29 Waxmonsky [64] | Cluster RCT | 384 | Providers at community based clinical practices | Unclear | Standard REP includes an intervention package consisting of an outline, a treatment manual and implementation guide, a standard training program, and as-needed technical assistance. Enhanced REP added customisation of the treatment manual and ongoing, proactive technical assistance from internal and external facilitators. | Group face to face with phone support |
| 30 Weinstein [41] | RCT (2x2 factorial design) | 400 | Women and their live born children | Trained counsellors | The interventions utilised either brief Motivational Interviewing  (MI) or traditional Health Education (HE) to provide oral health education, assist women to adopt behaviours associated with optimal oral health, and to seek professional dental care for themselves and their young children. | Individual, face to face |
| 31 Yamada [42] | Case study | Unclear | Council members and the health care professionals  employed in the NICU | A research  practice council in the NICU | Using knowledge transfer strategies to improve use of pain management strategies in hospitalised infants in neo-natal ICU | Group, face to face |
| 32 Yates [43] | RCT | 68 | Adult CABG patients and spouses participating in Cardiac Rehabilitation (CR) | Health professional delivering CR | Patients in both groups participated in the full CR program (comprehensive risk reduction, exercise sessions, and educational classes). Spouses/partners in the PaTH intervention group attended CR with the patient and participated in exercise sessions and educational classes to make the same positive changes in exercise and diet (Therapeutic Lifestyle Change [TLC] Diet recommended by the American Heart Association). Spouses in the usual care group were invited to attend the educational sessions that were part of the CR program. | Group, Face to face |
| 33 Zauszniewski [45] | Pilot (four groups) | 80 | Grandmothers who were raising grandchildren | Graduate nursing  or social work students | Personal and social resourcefulness skills training. | Individual, face to face |
| 34 Arends [68] | Cluster RCT | 212 | Workers aged between 18-63 years, diagnosed with a common mental disorder. | Occupational Physicians | Evidence-based guideline directed at structuring physicians’ treatment to help sick-listed workers with mental health problems to return to work, using strategies such as problem-solving. | Individual, face to face |
| 35 Bjelland [59] | Cluster RCT | 1465 | 11-12 year olds | Unclear | Intervention aimed at reducing intake of sugar- sweetened beverages and sedentary behaviour in adolescent school children. | Unclear |
| 36 Boschman [60] | Controlled trial (multi-site, non randomised) | 899 | Construction workers | Unclear | Intervention aimed at detecting signs of work-related health problems, reduced work capacity and/or reduced work functioning. | Individual, face to face |
| 37 Branscum [26] | Cluster RCT | 12 | YMCA-sponsored after school programs | Unclear | Knowledge and theory-based childhood obesity prevention intervention implemented in after-school programs. The knowledge-based intervention chose program activities to mediate behaviour change solely based on building awareness and knowledge, such as being aware of the recommended number of servings of fruits and vegetables. The theory- based intervention used theory-oriented program activities to mediate behaviour change such as taking small achievable steps for learning and mastering new skills. Both interventions also included aspects of making and reading comic books “Comics for Health.” | Group, face to face |
| 38 Brice [27] | Unclear | 226 | Families with recent live births | Emergency Medical Services staff | Infant and child safety focused intervention targeting ﬁre risks, water temperature, electricity, crib hazards, and ﬁrearms, as well as potential injuries associated with stairways, pools, and cars. Intervention strategies included the home safety assessment, one-on-one education and counselling, on-site home modiﬁcations, further recommendations, and referrals. | Individual, face to face |
| 39 Broekhuizen [46] | RCT | 181 | Individuals with familial hypercholesterolemia | unclear | Tailored lifestyle intervention aiming to reduce cardiovascular disease (CVD) risk by promoting a healthy lifestyle. Included: improving awareness of CVD risk, motivational interviewing, and computer-tailored lifestyle advice. | Individual face to face, computer, telephone |
| 40 Coffeng [74] | RCT | 363 | Employees | Team leaders | Group motivational interviewing combined with environmental changes to the physical workplace. | Group, face to face |
| 41 Cosgrove [49] | Pre-post (multi-site) | 57 | Patients with a primary diagnosis of COPD | Health professionals involved in delivery of pulmonary rehabilitation education sessions | Pulmonary rehabilitation programme that provides patients with disease-specific information and teaches self-management skills through the practical application of activities. Includes: educational materials and resources for both health professionals and patients). | Group, face to face |
| 42 Devine [69] | Pilot (multi-site, pre-post) | 226 | Female employees | Community nutrition professionals and worksite leaders | Locally adapted obesity prevention intervention involving goal setting, self-monitoring, modelling, and feedback on behaviour. | Individual, Web-assisted |
| 43 Fagan [62] | Cluster RCT | 12 | Youth communities | Unclear | The Communities That Care (CTC) operating system provides a planned and structured framework for diverse community partners to utilise advances in prevention science. Includes:, (a) assessing community readiness to undertake collaborative prevention efforts; (b) forming diverse and representative prevention coalitions); (c) using community-level epidemiologic data to assess prevention needs; (d) choosing evidence-based prevention policies, practices, and programs and (e) implementing new innovations with fidelity. | Group, face to face |
| 44 Gitlin [70] | Unclear | 41 | Caregivers for patients with dementia | Occupational therapists | Occupational therapists assess specific needs, concerns, and challenges of caregivers, the physical and social environment, caregiver management approaches, and dementia patient functionality. Involves environmental simplification, communication, task simplification, engaging patient in activities, and stress reduction, and five key treatment principles: client centered; problem solving; tailoring; action-oriented and cultural relevance. | Individual, face to face |
| 45 Goenka [30] | Cluster RCT | 5564 | Adolescent Students(6^th^ and 8^th^ Grade) | Teachers/Peers | Intervention involving multiple education sessions, school posters, and parent postcards focused on imparting behavioral skills and contextual knowledge to decrease children’s susceptibility to taking up tobacco in the future. | Individual + group, in person + posters+ postcards to take home |
| 46 Jonkers [52] | RCT (pragmatic) | 183 | Chronically ill elderly patients | Nurses | Minimal psychological intervention to reduce depression in chronically ill elderly persons involving self-monitoring, exploration of links between cognition, mood and behaviour, and action-planning. | Individual, face to face |
| 47 Lee-Kwan [71] | Pilot (8 groups) | 101 | Customers of restaurants serving unhealthy foods in deprived areas | Public health academics via provision of menu boards and posters in restaurants | A culturally appropriate health eating health promotion intervention in restaurants serving foods high in calories in low-income urban areas. | Individual, face to face |
| 48 Lisha[76] | Cluster RCT (3 groups) | 1426 | Adolescent high school Students | Four female health educators | A drug prevention programme, with and without combined motivation interviewing. | Group, face to face |
| 49 McCreary [53] | Pre-post (multi-site) | 294 | HIV patients | Volunteer peer leaders | The six-session intervention was delivered to small groups of 10–12 participants by 85 trained volunteer peer leaders working in pairs | Group |
| 50 Nakkash [55] | RCT | 196 | Currently married women, aged 18-49, reporting symptoms of medically unexplained vaginal discharge and low to moderate common mental disorders | Unclear | Psychosocial intervention package targeting the reporting of medically unexplained vaginal discharge and common mental disorders (depression and/or anxiety). Involves progressive muscle relaxation/ guided imagery exercises and weekly structured support groups. | Group |
| 51 Naven [84] | Unclear | 243 | Health visitors | NHS Health boards | Distribution programmes involved the distribution of free fluoride toothpaste and a toothbrush to all children in Scotland at the age of 8 months, and targeted distribution to 'at risk' children aged 1-3 years in areas of deprivation | Unclear |
| 52 Pbert [72] | Cluster RCT | 2710 | Adolescent (13-17years) smokers/ non-smokers/ former smokers | Paediatric providers | Smoking prevention and cessation intervention tailored to the stage of smoking acquisition of adolescents combined, with peer counselling focusing on the social aspects of smoking and development of the ability to resist social pressures to smoking. | Individual, face to face |
| 53 Potter [32] | Unclear | 660 | Students | Programme staff | Increase children’s exposure to a variety of fruit and vegetables by distributing free fresh or dried fruit and fresh vegetable snacks to all students during the school day. Teachers and school staff were allowed to eat the snacks to serve as role models. Nutrition education and promotion activities were encouraged but not required. | Group, face to face |
| 54 Skara [38] | Cluster RCT | 2331 | Adolescent high school students | Trained project health educators and  regular classroom teachers | Combined cognitive perception information and behavioural skills curriculum in a high school to prevent drug abuse. | Group, face to face |
| 55 Teel [40] | Pilot (single group, pre-post) | 6 | Older spouse caregivers of individuals with dementia | Advanced practice nurses | Intervention targeting healthy habits, self-esteem, communication, and self-care strategies in older adults. Included practicing healthy habits, building self-esteem, focusing on the positive, avoiding role overload, communicating, and building meaning. Specific self-care strategies were explored in the context of an individual’s experiences, relationships, and condition. | Individual, Telephone |

Note: Papers 1-33 are were identified with the forward citation search and papers 34 to 55 were identified with the electronic database search.
