# Supplementary material for: Methods used to address fidelity of receipt in health intervention research: a citation analysis and systematic review
Source: BMC Health Serv Res. 2016 Nov 18;16:663. doi: 10.1186/s12913-016-1904-6 (PMC5116196; doi:10.1186/s12913-016-1904-6)
Supplement: Additional file 2: — Details on methods used to assess and enhance receipt. (DOCX 91.1 kb) [file 12913_2016_1904_MOESM2_ESM.docx]

**Additional file 2.** Details on methods used to assess and enhance receipt

| References  (first author) | Guiding fidelity frame-work | Type of receipt addressed | | | | | | Method of data collection | | | % of sample | Sub-sample selection method | Timing of measurement | Reliability/  validity |
| --- | --- | --- | --- | --- | --- | --- | --- | --- | --- | --- | --- | --- | --- | --- |
|  |  | BCC definitions of receipt | | | | |  | Objective measure | Subjective measure | |  |  |  |  |
|  |  | Under- standing | | Perform-ance  of skill | | Multi-cultural factors | Other |  | Deliverer | Recipient |  |  |  |  |
|  |  | Assess | Enhance | Assess | Enhance |  |  |  |  |  |  |  |  |  |
| 1Asenlof [44] | BCC |  |  |  | ✓ |  | Intervention content | 1. Records of intervention sessions collected  2.Participants' individual working sheets collected to examine content received |  |  | 100% | NA | 1&2.During intervention (after intervention sessions) | NR |
| 2Battaglia [65] | BCC | ✓ |  | ✓ |  |  | Satisfaction |  |  | Self-report by participants of satisfaction (survey on satisfaction towards intervention curriculum and counselling; included questions on understandability) | 64% | Remaining participants withdrew | Unclear NR | Cronbach’s alpha for satisfaction with motivational interviewing measure |
| 3 Blaakman [66] | Linnan and Steckler, 2002 |  |  |  |  |  | Engagement  Satisfaction |  | 1. Nurses’ field notes and their review of audiotapes | 2. Self-reported satisfaction (questionnaire) | 80% | self-selection (missing data) | 1. During intervention  2.Post intervention | NR |
| 4 Black [25] | BCC | ✓ |  |  |  | ✓ | Behavioural change |  | 1.Documentation in care plan of participants' understanding and behavioural changes | 2.Verbal confirmation of understanding  3.Self-monitoring of behaviour change as a result of intervention | 100% | NA | 1,2 & 3. During intervention (at each encounter) | NR |
| 5 Bruckenthal [47] | BCC | ✓ |  | ✓ | ✓ |  | Homework completion |  |  | Review of participants' demonstration/practice of skills | 100% | NA | During intervention | NR |
| 6 Carpenter [48] | BCC | ✓ | ✓ | ✓ | ✓ |  | Successful attempts to contact participants  Availability of hardware to play intervention materials    Accepta-bility | 1. Number of participants not reached  2.Number of participants requiring media player to play intervention materials  3.Assessment of participants' ability to complete practice log | 4.Observation of participants' demonstration of intervention targeted behaviours (supported by pneumotrace data) | 5. Self-report of acceptability  (item in questionnaire capturing self-reported difficulties in breathing) | 1,2,3, & 5.100%  4. A subset of participants | 1,2,3, & 5. NA  4. Subset of people were those in one of the intervention groups (CD group) | 1. Three day follow-up call  2.During intervention  3. During intervention (at 2 weeks)  4. During and post-intervention (at 2, 8, and 16 weeks).  5. Post-intervention (at 8 and 16 weeks) | Cronbach’s alpha not computed for accepta-bility questionnaire because individual items examined |
| 7 Chee [28] | Lichstein et al 1994, TIM |  |  |  |  |  | Contacts with participants  Problem areas addressed in intervention |  | 1. Records of contacts 2. Records of problem areas addressed |  | 100% | NA | 1&2 During intervention  (at every session) | NR |
| 8 Culloty [75] | BCC | ✓^†^ |  | ✓ |  |  | Accepta-bility |  | 1.Direct observations (video-taped) of intervention sessions rated by evaluator against Process Evaluation of Training and Supervision (PETS) form | 2. Training Acceptability Rating Scale (TARS) questionnaire  3. Focus group interviews (1/7 questions on receipt) | 1. 33%,  2 &3. 100% | 1.NR for direct observations  2&3. NA | 1. During intervention (day 2 of 3)  2.Post-intervention  3. Focus group timing NR | Adequate psychometric properties reported (TARS, PETS) |
| 9 Delaney [61] | BCC | ✓ |  |  |  |  | Self-efficacy to perform skills targeted by intervention  Attitude following workshop |  |  | Self-report (questionnaires) by participants of knowledge, self-efficacy, and attitude in relation to intervention content | 100% | NA | pre and post intervention | Face and content validity of self-developed survey established by consensus |
| 10 Dyas [50] | BCC | ✓ |  |  |  |  | Intervention received |  | 1.Individual interviews on experiences using intervention | 2.Individual interviews on intervention received and understanding | 1. 100%  2. 30% | 2.By invitation (no further details) | 1&2.During intervention (midway through intervention period) | NR |
| 11 Eaton [51] | BCC |  |  | ✓ |  |  | Use of (Web) materials | 1. Website monitoring of chapter completion |  | 2. Self-reported computer skills (one survey item) | 100% | NA | 1. During intervention  2. Before intervention | NR |
| 12 Ford [29] | BCC | ✓ |  |  |  | ✓ |  |  |  | Individual questionnaire items on participants' understanding of and knowledge gain from the intervention | 100% | NA | Post-intervention | NR |
| 13 Kilanowski [31] | BCC | ✓ |  |  |  | ✓ | Attendance | 1.Attendance log |  | 2.Self-report of knowledge of nutrition/physical activity (CATCH questionnaire) | 1 & 2.  100% | NA | 1. During intervention (at all sessions  2.Pre and post | Psychometric properties of CATCH reported |
| 14 Michie [54] | BCC |  |  | ✓ |  |  | Behaviour change and/or maintenance |  |  | Self-report (audio-taped) of behaviour change and/or maintenance | Subsample (n=27) of 365 total sample size* | Purposive sampling of 52 participants, of which 27 had complete audiotape data* | During intervention | Analysis of transcripts verified by two coders |
| 15 Millear [77] | NR |  |  |  |  |  | Receptivity to carrying out intervention skills |  |  | Self-report by participant of whether intervention skills could be implemented in their daily lives (items part of evaluation questionnaire) | 100% | NA | Post-intervention (at 3 months and at 9 months) | NR |
| 16 Minnick [67] | Lichstein et al 1994, TIM | ✓ |  |  |  |  | Accuracy of recall of intervention content (comparison of participants' recall with deliverers' recall)  Uptake of practices taught |  | 1.Intervention deliverers' recall of intervention coaching activities  (reports and interviews) | 2.Participant recall of intervention coaching activities (reports and interviews)  3.Interviews with participants on uptake of practices taught and plans to sustain them | 100% | NA | 1. During intervention (daily or weekly reports/interviews)  2 & 3. Post-intervention interviews with participants | NR |
| 17 Pretzer-Aboff [33] | BCC | ✓ |  | ✓ |  |  |  |  | Direct observations of participants |  | 100% | NA | During intervention | NR |
| 18 Resnick [36] | BCC | ✓ |  | ✓ |  |  | Perceived effects of exposure to intervention |  |  | Focus group meeting with participants | 100% | NA | Post-intervention | NR |
| 19 Resnick [37] | BCC | ✓ |  |  |  |  |  |  |  | Knowledge questionnaire (Knowledge of Function-Focused Care Activities, KFFCA) | 100% | NA | Pre and post intervention (at 12 months) | Psychometric properties of KFFCA reported |
| 20 Resnick [35] | BCC | ✓ |  | ✓ |  |  |  |  |  | Knowledge questionnaire | 100% | NA | Pre and post intervention | NR |
| 21 Resnick [34] | BCC | ✓ |  | ✓ |  |  |  |  | Direct observations of participants by evaluator (use of checklist during observations) |  | < 100 %  (selection of 20 sessions, details NR) | Random | During intervention sessions | NR |
| 22 Resnick [56] | BCC | ✓ | ✓ | ✓ | ✓ |  | Attendance | 1.Attendance log | 2.Direct observation by deliverer of participants' verbal understanding and performance of skills against a checklist |  | 100% | NA | During intervention sessions | NR |
| 23 Robb [57] | BCC | ✓ | ✓ | ✓ | ✓ |  | Engagement |  | 1.Active questioning to assess understanding  2. Observation of participants' and rating of behavioural indicators of participant engagement on checklist |  | 100% | NA | During intervention sessions | NR |
| 24 Robbins [73] | BCC | ✓ |  |  |  |  | Attendance  Engagement | 1.Attendance logs | 2.Audio-recordings of deliverers' counselling sessions and evaluation by research team against checklist items on participant understanding and engagement |  | 1. 100%  2. 9% (3 of a total of 32 audiotapes sampled every other month) | 1. NA  2.Random | 1. During intervention (all sessions)  2.During intervention (all sessions audio taped) | NR |
| 25 Shaw [63] | BCC |  |  |  |  |  | Acceptability |  |  | Self-report by participants of acceptability (semi-structured telephone interview) | 88% | Data presented for subgroup with outcome data | Post-intervention (At 1 month post baseline) | Half of interviews double-coded; disagreements discussed |
| 26 Smith [58] | BCC |  |  |  |  |  | Attendance | Attendance logs |  |  | 100% | NA | During intervention sessions | NR |
| 27 Stevens [39] | Lichstein et al 1994, TIM | ✓ |  |  | ✓ |  | Active participation in workshop exercises and discussions  Receipt of written intervention materials  Feedback on workshop |  | 1.Notes and comments by research staff on observation of sessions | 2. Confirmation of receipt of materials from intervention sites and of comprehension of material  3. Self-report in feedback evaluation forms (goal and objectives, skill provided for rehabilitation teams, success of workshop) | 100% | NA | 1. During intervention sessions  2. During intervention  3. Post-intervention | NR |
| 28 Teri [78,78] | BCC | ✓ |  | ✓ |  |  |  |  | Observation of staff during role play workshop sessions (rated against checklist to assess knowledge, understanding and skill development) |  | 100% | NA | During intervention (recorded following each intervention session) | NR |
| 29 Waxmonsky [64] | BCC |  |  |  |  | ✓ | Attendance, Contacts | 1.Attendance logs | 2.Records kept of number of length of phone contacts |  | 1&2. 100% | NA | 1.During intervention sessions  2.During intervention | NR |
| 30 Weinstein [41] | BCC |  | ✓ |  | ✓ |  | Satisfaction |  | According to protocol, use of standardised protocols/manuals to enhance participants' understanding and use of skills (not assessed) | Self-report by participants in a feedback questionnaire  According to protocol, use of simple language to enhance understanding(not assessed) | 100% | NA | Post- intervention (after each of the 2 intervention period) | NR |
| 31 Yamada [42] | BCC |  |  |  |  |  | Acceptability |  |  | 1.Self-report of usefulness of implemented intervention strategies (checklist)  2.Barriers and facilitators to implementation of intervention strategies (data from meetings) | 100% | NA | 1.Post- intervention cycle  2.During intervention | Content validity reported and discusses construct validity of checklist developed |
| 32 Yates [43] | BCC |  |  |  |  |  | Attendance | Attendance logs |  |  | 100% | NA | During intervention | NR |
| 33 Zauszniewski [45] | BCC | ✓ |  | ✓ |  |  | Use of skills learnt during intervention |  |  | 1.Self-report of resourcefulness skills learnt and used (in daily journals or voice recordings)  2.Self-report of use of resourcefulness skills (questionnaire) | 100% | NA | 1.During the intervention  2. Post-intervention | Daily journals/recordings were coded by a coder blinded to group allocation  Confirmatory Factor Analysis, Cronbach alpha for questionnaire |
| 34 Arends [68] | Linnan and Steckler 2002 |  |  |  |  |  | Intervention components completed  Intervention content received |  | 1.Self-report by physician of number of assignments completed by patient (questionnaire) | 2.Self-report by patient of number of assignments completed (questionnaire)  3.Self-report by patient of topics discussed (checklist to complete) | 100% | NA | 1,2 & 3.During intervention (at 3 month follow-up) | NR |
| 35 Bjelland [59] | Saunders et al. 2005 |  |  |  |  |  | Satisfaction  Exposure  Receipt of intervention materials |  |  | 1. Parental self-report of awareness of intervention components,  receipt of and exposure to intervention materials, satisfaction (questionnaire) | 100% | NA | During intervention (at 8 months follow-up) | NR |
| 36 Boschman [60] | Linnan and Steckler, 2002 | ✓ |  |  |  |  | Recall of intervention-related advice  Intention to act on intervention advice |  |  | Self-report by participant (questionnaire) | 100% | NA | Post-intervention (immediately after and 3 months post-intervention) | NR |
| 37 Branscum [26] | Saunders et al. (2005) |  |  |  |  |  | Feasibility  Acceptability |  | Self-report by staff members of program feasibility and acceptability to participants  (questionnaire) |  | 100% | NA | Post-intervention | Staff members initially blind to program implemented |
| 38 Brice [27] | Linnan and Steckler, 2002 |  |  |  |  | ✓ | Satisfaction |  |  | Self-report by participants (survey) | 100% | NA | During intervention (12 months post baseline) | NR |
| 39 Broekhuizen [46] | Glasgow et al.,2007;Linnan and Steckler (2002),  Saunders et al. (2005) |  |  |  |  |  | Use of (Web) materials | Logins  Website monitoring of modules completed |  |  | 100% | NA | During intervention | NR |
| 40 Coffeng [74] | Linnan and Steckler, 2002 |  |  |  |  |  | Attendance  Use of intervention components |  |  | Self-report of attendance of intervention sessions and use of intervention components (questionnaire) | 100% | NA | Post -intervention | NR |
| 41 Cosgrove [49] | Saunders et al. 2005 | ✓ |  |  |  |  | Acceptability  Satisfaction |  | 1.Self-report by deliverer of acceptability of intervention (questionnaire) | 2.Self-report by patient of acceptability of intervention (questionnaire)  3.Self-report by patient of satisfaction with educational component (questionnaire) | 100% | NA | 1&2. During intervention (at each session)  3. Post-intervention | NR |
| 42 Devine [69] | Linnan and Steckler, 2002 |  |  |  |  |  | Intervention content received |  | 1.Self-report by deliverers' on experiences with intervention and influencing contextual factors via semi-structured interviews and focus groups (score assigned to sites as indicator of portion of intervention elements received out of those possible) |  | Representative sub-sample of site staff in 4 of 5 sites (proportion of total site staff unclear) | Non-random (no further details provided) | Post-intervention (3-6 months after intervention) | Scores for dose received assigned independently by two researchers (kappa given) |
| 43 Fagan [62] | Dusenbury et al (2005) | ✓ |  |  |  |  | Responsiveness |  | Self-report by deliverers of participants' understanding and participation (2 item questionnaire) |  | 100% | NA | During intervention (years 2 through to 5 of study) | NR |
| 44 Gitlin [70] | Glasgow et al.,2007 | ✓ |  | ✓ |  | ✓ | Adequacy on number of sessions received  Treatment received with respect |  |  | 1.Self-report of receipt (questionnaire with items on adequacy of number of sessions received, treated with respect, skills learnt and understanding) | 50% | Self-selected  (missing data) | Post-intervention | NR |
| 45 Goenka [30] | Saunders et al., 2005 | ✓ |  |  |  | ✓ | Enjoyment and communication skills during intervention delivery  Confidence in using intervention materials and principles  Students' absorption, engagement, participation, ease of use of program materials |  | 1.Self report by deliverers of own enjoyment in teaching, communication skills with participants, ease of use of handbook materials, confidence in using intervention strategies (questionnaire)  2.Self-report by deliverers of participants' enjoyment, ease of use of materials, participation and absorption (questionnaire)* |  | 100% | Unclear | 1 & 2.During intervention (after each session) | NR |
| 46 Jonkers [52] | Linnan and Steckler, 2002  Baranowski and Stables, 2000 |  |  |  |  |  | Engagement  Intention to implement intervention  Satisfaction  Adherence to commitments  made |  | 1.Self-report by nurse of patient's ability to understand and implement intervention principles (questionnaire)  2.Self-report by nurses of patient's adherence to previous commitments (checklist)  3. Self-report by nurses of satisfaction with intervention (group interview) | 4.Self-report by patient of intention to implement intervention in daily lives (questionnaire)  5. Self-report by patient of satisfaction with intervention (questionnaire) | 100% | NA | 1 & 2. During intervention (at each intervention contact)  3, 4, & 5. Post-intervention | NR |
| 47 Lee-Kwan [71] | Linnan and Steckler, 2002 |  |  | ✓ |  | ✓ | Exposure to intervention materials  Behavioural change following exposure |  |  | Self-report by participants of whether they had seen intervention materials (menu boards/posters) and impact on food purchasing behaviours (survey) | 20% | Every 5^th^ customer approached for assessment | Post-intervention | NR |
| 48 Lisha[76] | Dane & Schneider, 1998) |  |  |  |  |  | Attendance | Attendance log*** |  |  | 100% | NA | During intervention (each sessions) | NR |
| 49 McCreary [53] | Linnan and Steckler, 2002 |  |  |  |  |  | Engagement in group sessions |  | Observations of participants' answers and rating of participants' engagement in group sessions against checklist items (relative to readiness to answer, responses to other participants, and focus on presentation)  2.Self-report (qualitative comments) by deliverers based on observations |  | < 100%  294 (64%) group sessions observed out of a total estimated to be 459 sessions^£^ | Convenience sample of group sessions | 1 & 2. During intervention (every 30 minutes during group session | Cronbach’s alpha reported for the rating scale used to assess participant engagement |
| 50 Nakkash [55] | Linnan and Steckler, 2002 |  |  |  |  |  | Satisfaction  Level of involvement |  | 1.Self-report by deliverers of participants' participation and involvement in intervention sessions (questionnaire) | 2.Self-report by participant of satisfaction with intervention sessions (questionnaire) | 100% | NA | 1 & 2. During intervention (completed after each intervention session) | NR |
| 51 Naven [84] | NR |  |  |  |  |  | Receipt of information on intervention requirements |  | Self-report by participating sites of whether intervention requirements  were received (item in survey) |  | <100%; all health visitors in 3 NHS Boards, but only a subsample of NHS Board 4. | NHS Board 4 subset of health visitors was randomly selected | Unclear (during or post-intervention) | NR |
| 52 Pbert [72] | NR |  |  |  |  |  | Occurrence of possible intervention steps |  |  | Self-report by patient of intervention steps followed by deliverer(survey) | 100% | NA | Post-intervention | NR |
| 53 Potter [32] | NR |  |  |  |  |  | Reactions to program |  |  | 1.Self-report by students and parents of reactions to program (separate focus groups)  2.Self-report by school staff of reactions to program (survey and in person interviews) | <100%; 5 (20%) of 25 schools awarded program funding were included in evaluation  Proportion of respondents from these 5 schools included in the evaluation is unclear. | Specific eligibility criteria set to select 5/25 school;  Focus groups: convenience sample  Remaining: selection method unclear | Post-intervention | NR |
| 54 Skara [38] | Dane & Schneider, 1998 Dusenbury et al., 2003) |  |  |  |  |  | Responsiveness to program |  |  | Self-report by students of responsiveness to program (questionnaire) | 100% | NA | Post-intervention | NR |
| 55 Teel [40] | Lichstein et al 1994, TIM | ✓ |  |  |  |  | Adequacy of communication methods used in intervention |  |  | 1. Self-report on helpfulness/usefulness of intervention to assess understanding of intervention content (interview data)  2.Self-report by participant on adequacy of communication method used in intervention sessions (questionnaire) | 100% | NA | 1& 2. Post intervention | NR |

*Abbreviations:* BCC- Behaviour Change Consortium; TIM- Treatment Implementation Model; Des- Design; Del-Delivery; T-training; En-Enactement; ✓ Included in paper

*Notes*: Papers 1-33 are from the forward citation searching and papers 34 to 55 are from the database searches. A measure of knowledge was considered to assess understanding. Attendance logs, and tracking of contacts/interactions with study participants have usually been classified as objective measures of receipt given they are less prone to being influenced by perceptions/biases. It is acknowledged however that these assessments of receipt are based on reports made by the research team/intervention deliverers. Where direct observations were carried out and rated/assessed against a checklist by the interventionist, they are listed under 'Subjective report-provider'. Where they are listed as the 'Objective' assessment method column, it is because they were evaluated by the research team (and usually recorded for this purpose)

^†^ Authors report that amongst others, item 7 of the TARS was used to assess receipt. We searched for the TARS tool online. It was found to assess participants' understanding of the intervention (<http://www.seattleimplementation.org/wp-content/uploads/2012/10/M_Milne_TARS-2.pdf>)

* details found in a paper referenced in the primary study in question

**This paper provided information relative to dose received during implementers' training to deliver the intervention, and information relative to dose received during delivery of the health intervention in real-life settings (across schools). Only data on the latter appears in this table.***A measure of participant responsiveness (self-report of participants' responses to the program in a questionnaire) was included in this paper but was considered by authors to be separate to dose received, therefore it is not included in this table.

In type of receipt assessed, assessment of skill performance can be carried out on interventionists if it is conceptualised to be an indicator of intervention receipt by the authors (e.g. [75] the extent to which the intervention deliverer used facilitated learning methods during the intervention sessions is considered to be an indicator of intervention receipt)

^¥^ In some cases, methods to assess receipt include direct observations that are made by the evaluators/research team, rather than by intervention deliverers'. Where this is the case, it is specified.

In most cases, the proportion (%) of intervention group participants on which receipt was assessed refers to the intended proportion described in the methods sections of the papers. Where this was not reported/unclear, the proportion of the intervention group for the results were reported is indicated.

£Rough estimate calculated using information on total number of participants recruited n=4597 (with n=2242 from rural communities, n=1500 teenagers, n=855 hospital workers) and assumption that group sessions included 10 people (suggested to be 10-12 in paper)

Where information on psychometric properties was provided, this may have been in relation to data collected in the study or to previous research investigating psychometric properties.

Frameworks used and definitions of receipt:

-In Linnan and Steckler (2000), receipt is defined as 'the extent to which participants actively engage with, interact with, are receptive to, and/or use materials or recommended resources'

-In Saunders et al. (2005), receipt is broken down into dose received (exposure) defined as 'the extent to which participants actively engage with, interact with, are receptive to, and/or use materials or recommended resources', and dose received (satisfaction), defined as 'participant (primary and secondary audiences) satisfaction with program, interactions with staff and/or investigators.'

-In Lichstein et al (1994), receipt is defined as the accuracy of the participants' understanding of the intervention

- In Baranowski et al. (2000), receipt is conceptualised as reach, or receipt of, of materials by the target group

-In RE-AIM (Glasgow et al.,2007), receipt per se is not part of the framework. Concepts included in the framework and similar to receipt in other frameworks are: participation (i.e. participation rates), fidelity of implementation (data on implementation variations of different program components during the evaluation)

- Dusenbury et al. (2003; 2005) support Dane and Schneider's (1998) framework in which five components of fidelity are proposed and propose additional components to investigate. The component closest to receipt and common to both frameworks is participant responsiveness (no clear definition provided).
